# p53 oligomerization status as an indicator of sensitivity of p53-wildtype neuroblastomas to the combination of DNA damaging agent and Chk1 inhibitor

**DOI:** 10.1371/journal.pone.0263463

**Published:** 2022-02-10

**Authors:** Aime A. Levesque, Rebecca M. Pappalardo, Pawan Puli, Laura A. Enzor, Clara Angeles

**Affiliations:** Department of Biology, University of Hartford, West Hartford, Connecticut, United States of America; Virginia Commonwealth University, UNITED STATES

## Abstract

Neuroblastomas are one of the most common types of solid tumors in infants and children and are responsible for approximately 15% of childhood cancer deaths. Neuroblastomas rarely have mutations in p53, with less than 2% of NB containing mutations in p53, compared to up to 60% for other tumor classes. Previous studies on the therapeutic combination of a DNA damaging agent and checkpoint kinase 1 (Chk1) inhibitor have shown that DNA damage-induced cell cycle arrest can be specifically abrogated in p53-defective tumors. However, some p53-wildtype tumors have also been shown to be sensitive to this therapeutic combination, suggesting that these cells have other defects in the p53 response that can be exploited for therapeutic purposes. In the current study, we investigated the response to the combination of a DNA damaging agent (SN38) and a Chk1 inhibitor (UCN-01) of four p53-wildtype neuroblastoma cell lines: SK-N-SH, SH-SY5Y, SK-N-AS, and Lan-5. When the cells were treated with concentrations of SN38 ranging from 0–30 ng/ml, all four cell lines accumulated p53 which was phosphorylated on serines 15 and 20. However, only the SK-N-SH were found to activate p21^waf1^ and repress cyclin B. In order to assess sensitivity to UCN-01-mediated abrogation of cell cycle arrest, cell were treated with 10 ng/ml SN38 for 24 h, followed by 25 nM UCN-01 for 6 and 24 h. The SK-N-SH showed no sensitivity to UCN-01 treatment whereas the SH-SY5Y, SK-N-AS, and Lan-5 abrogated G_2_ arrest within 24 h. Our recent studies revealed that cells that are sensitive to checkpoint abrogation lack p53 dimers and tetramers, so we analyzed the oligomerization status of p53 in all four cell lines using glutaraldehyde crosslinking. The SK-N-SH cells possessed levels of p53 dimers and tetramers similar to what has previously been reported in p53-wildtype MCF10A cells. The SH-SY5Y, SK-N-AS, and Lan-5 however, had extremely low to undetectable levels of dimers and tetramers. Our study also showed no cytoplasmic accumulation of p53 in these cells contrary to some previous reports. The results of this study suggest that oligomerization status may serve as an indicator of sensitivity of p53-wildtype tumors to the therapeutic combination of DNA damaging agent and Chk1 inhibitor.

## Introduction

Neuroblastomas are one of the most common types of tumors in infants and children. It represents 8–10% of all tumors and 15% of all cancer-related deaths in children [1], and has a long term survival rate of less than 50% [2–6]. Neuroblastoma occurs at a rate of 10.2 cases/million children under the age of 15 years, with approximately 500 new cases diagnosed yearly [7]. Although, the incidence seems relatively low, and the 5 year survival rate has improved in the last 30 years, the severity of the disease and poor survival rate in the high risk group is a major concern [8].

The tumor suppressor protein, p53, is considered the guardian of the genome; it holds the key to induce cell cycle arrest and/or apoptosis in response to DNA damage. It is mutated in up to 60% of all human tumors, highlighting the central role it plays in preventing normal cells from becoming cancerous. In the absence of damage, p53 levels are kept low through its association with MDM2, an E3 ubiquitin ligase that promotes degradation of p53 by the proteosome [9]. If DNA damage is present at any point during the cell cycle, p53 is activated by phosphorylation at serine 15 by ATM and ATR protein kinases and at serine 20 by the checkpoint kinases Chk1 and Chk2 [10–12]. These phosphorylation events lead to the dissociation from MDM2, allowing p53 to stabilize, form oligomers and activate the transcription of a series of genes involved in regulation of the cell cycle, including p21^waf1^, GADD45, 14-3-3σ, and MDM2 [13]. The activation of ATM and ATR kinases also triggers a p53-independent pathway that consists of the phosphorylation and activation of Chk1, which inhibits the cdk/cyclin complex and halts the cell cycle in the S and G2 phases [14]. It has also been observed that p53 represses transcription of some target genes, including the mitosis promoting cyclin B [15], which further supports cell cycle arrest in response to DNA damage.

In the past few decades, there has been significant interest in the therapeutic combination of a DNA damaging agent and a Chk1 inhibitor [14, 16, 17]. Several early studies using this combination showed that p53 defective cells would abrogate DNA damage-induced cell cycle arrest and undergo a lethal mitosis, while p53-wildtype cell lines would not [18–23]. The use of this therapeutic combination has great promise in the treatment of p53 defective tumors, although clinical trials have yielded mixed results [17]. However, some p53-wildtype tumors have been found to be sensitive to this treatment as well [24], which would serve to broaden the spectrum of cancers that can be treated using this therapeutic combination.

Mutations in p53 are found in less than 2% of neuroblastomas, and most of those are found in relapsed neuroblastomas [25]. Multiple studies have investigated the possible inactivation of p53 in p53-wildtype neuroblastomas. Some studies have concluded that the DNA damage checkpoint response was attenuated in neuroblastomas due to cytoplasmic sequestration of p53 [26–28]. Furthermore, there is evidence that aberrant structural modifications or hyperubiqutylation are the cause for this cytoplasmic sequestration [29, 30]. However, this remains an area of some controversy, as other studies have shown that p53 is nuclear in neuroblastomas [31–33]. It has also been proposed that overexpression of MDM2 may be responsible for inactivation of p53 in p53-wildtype neuroblastomas [34].

In our previous studies, we investigated the sensitivity of a p53 wildype cell line as well as two isogenic derivatives that either overexpress the p53 oligomerization domain (MCF10A/OD) or overexpress an shRNA targeting p53 mRNA for degradation (MCF10A/Δp53) to the therapeutic combination of SN38 and UCN-01 [35]. As expected, the MCF10A cells were not sensitive to this treatment, while the MCF10A/Δp53 were. What was suprising was that the MCF10A/OD abrogated S but not G2 arrest, and failed to repress cyclin B, yet still activated p21^waf1^. Our subsequent studies showed that the MCF10A/OD cells formed p53 dimers, but failed to form tetramers [36], which suggests that dimers alone are sufficient for p53 transcriptional repression function, while tetramers are needed to transcriptional activation as has been previously observed [37, 38]. This observation led us to ask whether cell lines which have wildtype p53 may be sensitive to checkpoint abrogation due to a failure to form oligomers.

In the current study, we used p53-wildtype neuroblastomas, SK-N-SH, SH-SY5Y, SK-N-AS, and Lan-5, to test the hypothesis that cells with wildtype p53 that are sensitive to the combination of SN38 and UCN-01 will fail to form tetramers upon DNA damage. We observed that the SK-N-SH behaved as expected for a p53-wildtype cell line: they were insensitive to UCN-01, activated p21^waf1^, repressed cyclin B, and had normal levels of dimers and tetramers. The other three cell lines, however, were sensitive to UCN-01, failed to activate p21^waf1^ or repress cyclin B, and failed to form dimers or tetramers. This suggests that the oligomerization status of p53-wildtype tumors may serve as a valuable indicator of the likely effectiveness of the therapeutic combination of a DNA damaging agent and a Chk1 inhibitor.

## Materials and methods

### Chemicals

The DNA damaging agent used in this study was the topoisomerase-1 inhibitor SN38 (provided by Pfizer Global, Kalamazoo, MI, USA). The Chk1 inhibitor used in this study was 7-hydroxystaurosporine, or UCN-01 (provided by Dr. Edward Sausville, National Cancer Institute, Bethesda, MD, USA). For all SN38 treatments, cells were plated at 500,000 cells per well in 6 well plates and after 24 h, the media was removed and replaced with fresh media containing SN38 for 24 h. In procedures where UCN-01 treatment followed SN38 treatment, cells were treated with 10 ng/mL of SN38 for 24 h, after which the media containing SN38 was washed away using phosphate buffered saline (PBS) before the addition of fresh media with or without 25 nM UCN-01.

### Cell culture

The p53-wildtype neuroblastoma cell lines, SK-N-SH, SH-SY5Y, and SK-N-AS were obtained from American Type Culture Collection (Manassas, VA, USA), and the Lan-5 were donated by the Children’s Oncology group at the Texas Tech University Health Sciences Center (Lubbock, Texas, USA). The SK-N-SH, SH-SY5Y, and SK-N-AS were cultured in DMEM media supplemented with 10% Fetal Bovine Serum (FBS), penicillin (100 U/ml), streptomycin (100 mg/ml), and fungizone (0.25 mg/ml). The SH-SY5Y were weakly adherent and were grown on Corning Cell Bind plates and flasks (Corning, NY, USA) in order to aid cellular adhesion. The Lan-5 were cultured in RPMI-1640 media supplemented with 10% Fetal Bovine Serum (FBS), penicillin (100 U/ml), streptomycin (100 mg/ml), and fungizone (0.25 mg/ml). All cells were grown in a 5% CO_2_ incubator at 37°C.

### Immunoblotting

Cell lysates were prepared by washing cells with PBS and then harvesting in Laemmli sample buffer [39]. The harvested samples were boiled for 5 minutes before being stored at -20°C. Proteins in the cell lysate samples were separated by SDS-PAGE using 8% gels for most immunoblots, with the exception of 10% for p21 blots and 6% for glutaraldehyde crosslinking samples. The proteins were then transferred to PVDF membranes which were blocked in 5% milk in Tris-buffered saline containing 0.1% Tween 20 (TBST). To conserve antibody, the membranes were cut after transfer (at just below 37 kDa and just above 75 kDa for most blots, and just below 20 kDa and just above 37 kDa for p21 blots). The membrane was probed for the protein of interest using a specific primary antibody diluted in 5% milk in TBST and incubated in primary antibody overnight at 4°C [cyclin B1 (GNSI), p21 (C-19), p53 (DO-1) from Santa Cruz Biotechnology, Santa Cruz, CA, USA; β-actin, phospho-p53 ser15, phospho-p53 ser20 from Cell Signaling Technology, Beverly, MA, USA; RNA Polymerase II from Active Motif, Carlsbad, CA, USA]. The following day, the membranes were washed in TBST before incubation with an HRP-conjugated secondary antibody (BioRad, Hercules, CA, USA) for 1 h. The membrane was then washed with TBST and proteins were visualized using LumiGlo chemiluminescence substrate (KPL, Inc., Gaithersburg, MD, USA) followed by exposure to film. Film was hand-developed using Kodak GBX fixer and developer following the manufacturer’s protocol. For all experiments where multiple cell lines were analyzed for the same protein and could not fit on the same gel, samples were run in parallel on separate gels and the same exposure times were used.

### Cell cycle analysis

DNA content was assessed by flow cytometry as reported previously [35, 40]. Cyclin B levels were assessed by incubating fixed cells with a FITC-conjugated anti-cyclin B antibody (Pharmingen, San Diego, CA, USA) as previously described [20]. DNA content and cyclin B levels were determined by flow cytometry using a Becton Dickinson FACScan. For both the SN38 dose response and UCN-01 treatments, three trials were completed. Flow images contain representative images from one trial, while graphs contain data from three trials.

### Nuclear and cytoplamsic extracts

Cells were treated with 10 ng/ml SN38 for 24 h in order to activate p53. Cells were harvested in trypsin and nuclear and cytoplasmic extracts were then prepared using the NE-PER Nuclear and Cytoplasmic Extraction Reagents (Thermo Scientific, Waltham, MA, USA) following the manufacturer’s instructions. Laemmli sample buffer was added to each sample, and samples were then boiled for 5 min and analyzed by SDS-PAGE followed by immunoblot.

### Glutaraldehyde crosslinking

Glutaraldehyde crosslinking was performed as previously reported [36]. Cells were treated with 10 ng/ml SN38 for 24 h in order to activate p53. Cells were washed in phosphate buffered saline and then lysed in lysis buffer (10nM Tris pH 7,6, 140 nM NaCL, % NP40) supplemented with protease inhibitor cocktail (Sigma Aldrich, St. Louis, MO, USA), and phosphatase inhibitor cocktail (Sigma Aldrich). A glutaraldehyde solution was added to samples of lysate at final concentrations of 0.01 and 0.03%, and the samples were incubated at room temperature for 5 min. After addition of Laemmli sample buffer to each sample, the samples were boiled for 5 min and were analyzed by SDS PAGE followed by immunoblot.

### Statistical analysis

Statistical significance for the SN38 dose response was determined using a one-way analysis of variance (ANOVA). Statistical significance for the UCN-01 treatments was determined using a two-way ANOVA. In both cases, a Fisher LSD *post-hoc* test was utilized, and results were deemed to be statistically significant if the p-value was less than 0.05.

## Results

We have previously observed that p53-wildtype immortalized breast epithelial cells, MCF10A, arrest in the cell cycle upon treatment with SN38 in a concentration-dependent manner, and that this arrest cannot be abrogated by treatment with UCN-01, while cells lacking p53 (MCF10A/Δp53) will abrogate arrest [35]. Surprisingly, we have also observed that some cells with wildtype p53 will abrogate DNA damage-induced arrest when treated with UCN-01 [24]. In order to further understand the checkpoint response in p53-wildtype tumors, we now turn our attention to four neuroblastoma cell lines with wildtype p53: SK-N-SH, SH-SY5Y, SK-N-AS, and Lan-5.

**Fig 1:**
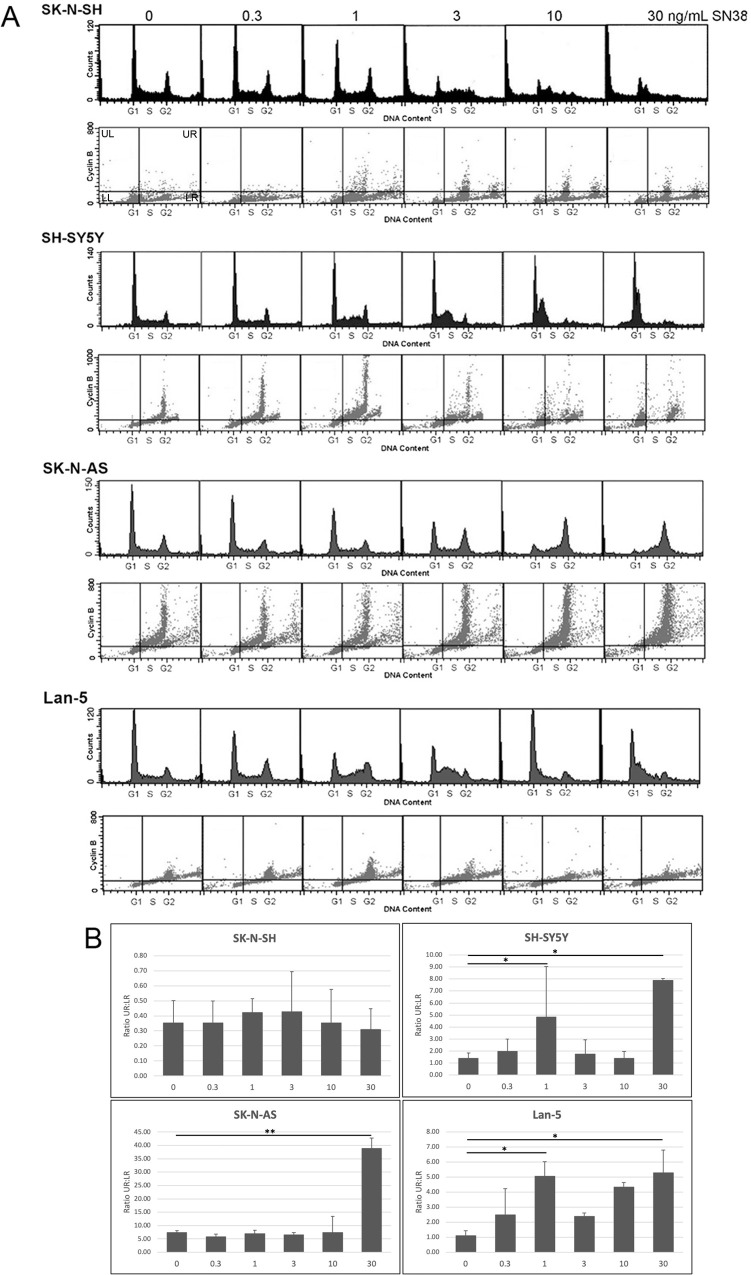
Impact of SN38 treatment on cell cycle distribution in SK-N-SH, SH-SY5Y, SK-N-AS, and Lan-5. Cells were incubated with 0–30 ng/ml SN38 for 24 h. The cells were then harvested, fixed, incubated with propidium iodide and FITC-conjugated anti-cylin B antibodies, and analyzed by two-dimensional flow cytometry (A). The first cyclin B panel for SK-N-SH indicates the four quadrants: upper left (UL), lower left (LL), upper right (UR), and lower right (LR). Bar graphs (B) show the ratio of cells in UR to LR for each treatment condition presented as mean ± SEM, n = 3 (*p<0.05, **p<0.001).

Changes in cell cycle distribution in p53-wildtype neuroblastoma cell lines SK-N-SH, SH-SY5Y, SK-N-AS, and Lan-5 in response to treatment with increasing concentrations of SN38 (0–30 ng/ml) was analyzed by flow cytometry. The SK-N-SH, SH-SY5Y, and Lan-5 arrested in G2 and S phase at lower SN38 concentrations and at G1 at the higher concentrations (Fig 1A). The SK-N-AS, however, appeared to arrest in G2 at the highest concentrations (Fig 1A). The sub-G1 cell populations observed in SK-N-SH cells at high SN38 concentrations suggest that these concentrations have increased levels of cytotoxicity in this cell line.

We have previously observed that cyclin B is repressed in a p53-dependent manner [24, 35]. In order to determine whether cyclin B expression was repressed in the neuroblastoma cells, we also investigated changes in the levels of cyclin B in response to increasing concentrations of SN38 by two-dimensional flow cytometry (Fig 1A). Since cyclin B is normally only expressed in cells in G2/M and the proportion of cells in G2/M varies greatly under different SN38 concentrations and among cell lines, we used the ratio of cells in the upper right (UR) to lower right (LR) quadrants in our analyses (these include cells in S as well as G2/M). Cells with high cyclin B will be in the UR while the cells with low cyclin B will be in the LR quadrant. A high ratio of UR:LR indicates that cyclin B is not repressed, while a low ratio indicates it is repressed. In SK-N-SH, cyclin B appears to remain repressed at all concentrations of SN38, with no significant change in the ratio of UR:LR at any concentration of SN38 compared to untreated control (Fig 1B). However, in the SH-SY5Y, SK-N-AS, and Lan5, there is a statistically significant increase in the ratio of UR:LR at the highest SN38 concentration compared to untreated control (Fig 1B). The increase in SK-N-AS is especially pronounced with a significance of P<0.001, but this is largely due to the fact that this cell line has the largest population of cells in G2 at that SN38 concentration (Fig 1A). The SH-SY5Y and Lan-5 also had a statistically significant increase in the ratio of UR:LR at the 1 ng/ml SN38 concentration (Fig 1B). These results suggest that cyclin B remains repressed in the SK-N-SH but not in the other three cell lines. The proportions of cells in all four quadrants (UR, LR, UL, LL) are included in S1 Fig.

**Fig 2:**
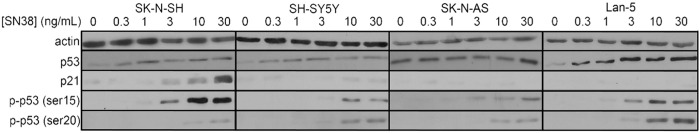
The impact of SN38 treatment on cell cycle regulatory proteins in SK-N-SH, SH-SY5Y, SK-N-AS, and Lan-5. Cells were incubated with 0–30 ng/ml SN38 for 24 h. Cells were harvested and lysates were analyzed by Western blot with the indicated antibodies. All samples for a given cell line blotted with a given antibody were run on the same gel. The same exposure times were used for different cell lines for the same antibody although they were not necessarily run on the same gel.

Changes in levels of p53 and p21^waf1^ in response to increasing concentrations of SN38 were analyzed using Western blot (Fig 2). The SK-N-SH, SH-SY5Y, SK-N-AS, and Lan-5 were all found to have p53 levels that increased with increasing SN38 concentrations while the actin loading control remained relatively stable (Fig 2). Activation of p53 was observed by blotting for p53 phosphorylated at serines 15 and 20. Importantly, DNA damage resulted in phosphorylation of p53 on serines 15 and 20 in all four cell lines (Fig 2). Levels of the cyclin-dependent kinase inhibitor p21^waf1^ increased in the SK-N-SH but not in the SH-SY5Y, SK-N-AS, or Lan-5 indicating a defect in p53 transcriptional activation function.

**Fig 3:**
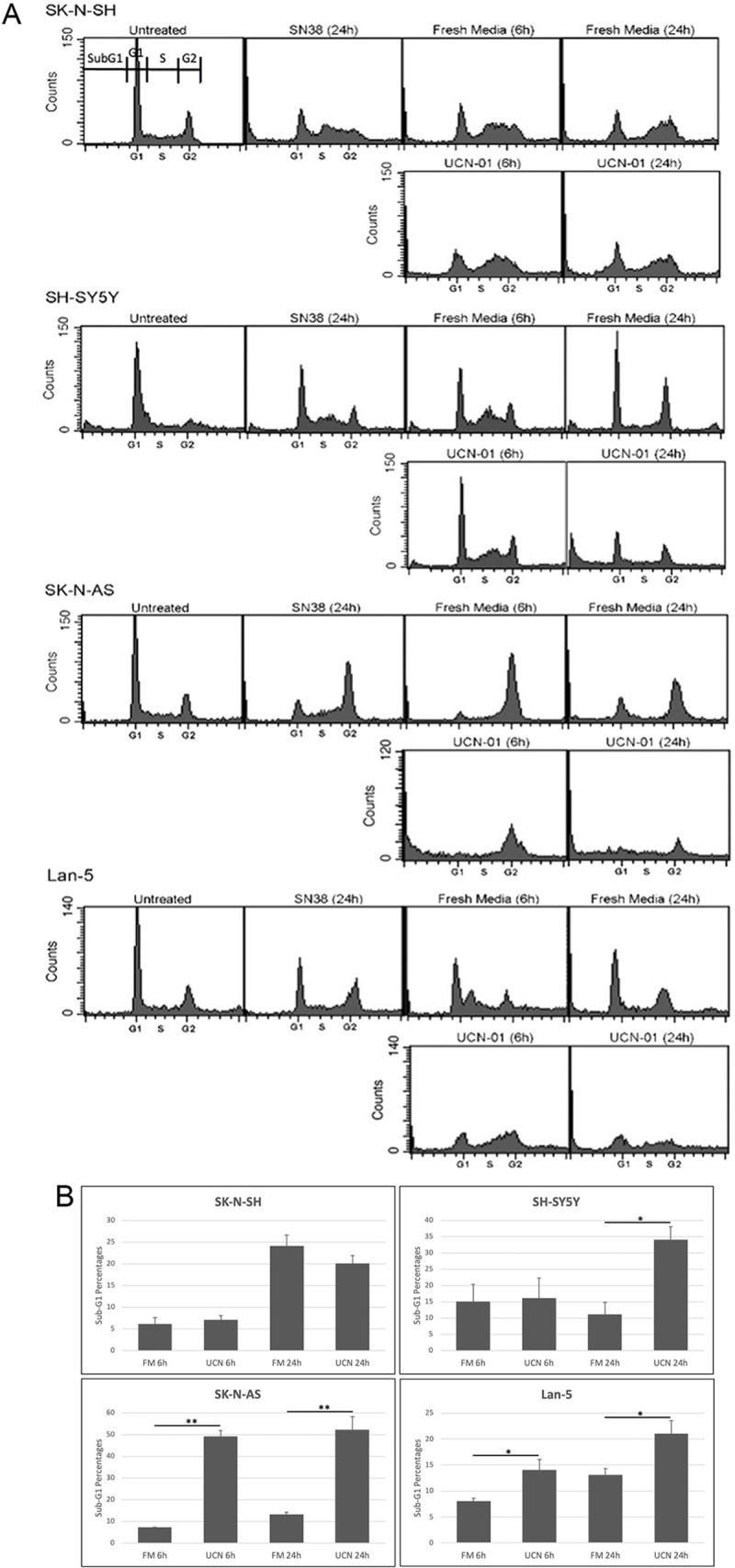
Response of SK-N-SH, SH-SY5Y, SK-N-AS, and Lan-5 to combination treatment with SN38 and UCN-01. Cells were incubated with 10 ng/ml SN38 for 24 h. The media was removed and was replaced with either fresh media or media containing 25 nM UCN-01 for an additional 6 and 24 h. The cells were harvested at the indicated times, fixed, stained with propidium iodide, and analyzed for DNA content by flow cytometry (A). The first panel for SK-N-SH indicates the four cell cycle stages measured: SubG1, G1, S, and G2/M. Bar graphs (B) represent percentage of cells in SubG1 population under indicated conditions presented as mean ± SEM, n = 3 (*p<0.05, **p<0.001).

In order to determine whether the two neuroblastoma cell lines were sensitive to checkpoint abrogation following treatment with UCN-01, cells were treated with 10 ng/ml of SN38 for 24 h to induce DNA damage, and then were incubated in fresh media with or without 25 nM UCN-01 for 6 and 24 h. Abrogation was defined as an increase in the population of SubG1 cells and only the SubG1 percentages are included in the statistical analysis (Fig 3B). The distribution of cells in all cell cycle stages (SubG1, G1, S, and G2/M) are included in S2 Fig. For SK-N-SH, the UCN01 treatment at 6 and 24 h appeared very similar to the fresh media treatment at those time points (Fig 3A) indicating that these cells were unaffected by the addition of UCN-01. Additionally, there was no statistically significant change in the percentage of SubG1 cells when comparing fresh media to UCN-01 treatments at each timepoint for SK-N-SH (Fig 3B).

For the SH-SY5Y there was not a significant difference in the SubG1 populations between the fresh media and UCN-01 treatments 6 h after UCN-01 (Fig 3B). At 24 h, however, a significant difference between the two groups was observed (Fig 3B). For both the SK-N-AS and SH-SY5Y there was a statistically significant difference between the fresh media and UCN-01 groups at both 6 and 24 h (Fig 3B). These results indicate that the SK-N-SH fail to abrogate arrest upon treatment with UCN-01, while the other three cell lines are sensitive to checkpoint abrogation upon treatment with UCN-01.

Collectively, these results revealed that SK-N-SH cells have fully functional p53 that was phosphorylated at serines 15 and 20 and activated expression of p21^waf1^ and repressed expression of cyclin B. These cells were insensitive to UCN-01-mediated checkpoint abrogation as would be expected for a p53-wildtype cell line. The SH-SY5Y, SK-N-AS, and Lan-5 cell lines also had p53 that was phosphorylated at serines 15 and 20 yet did not activate p21^waf1^ expression and failed to repress cyclin B. These cells were sensitive to UCN-01 mediated checkpoint abrogation. We considered two possible explanations: that p53 was cytoplasmically sequestered in the SH-SY5Y, SK-N-AS, and Lan-5, or that they lacked p53 oligomers.

**Fig 4:**
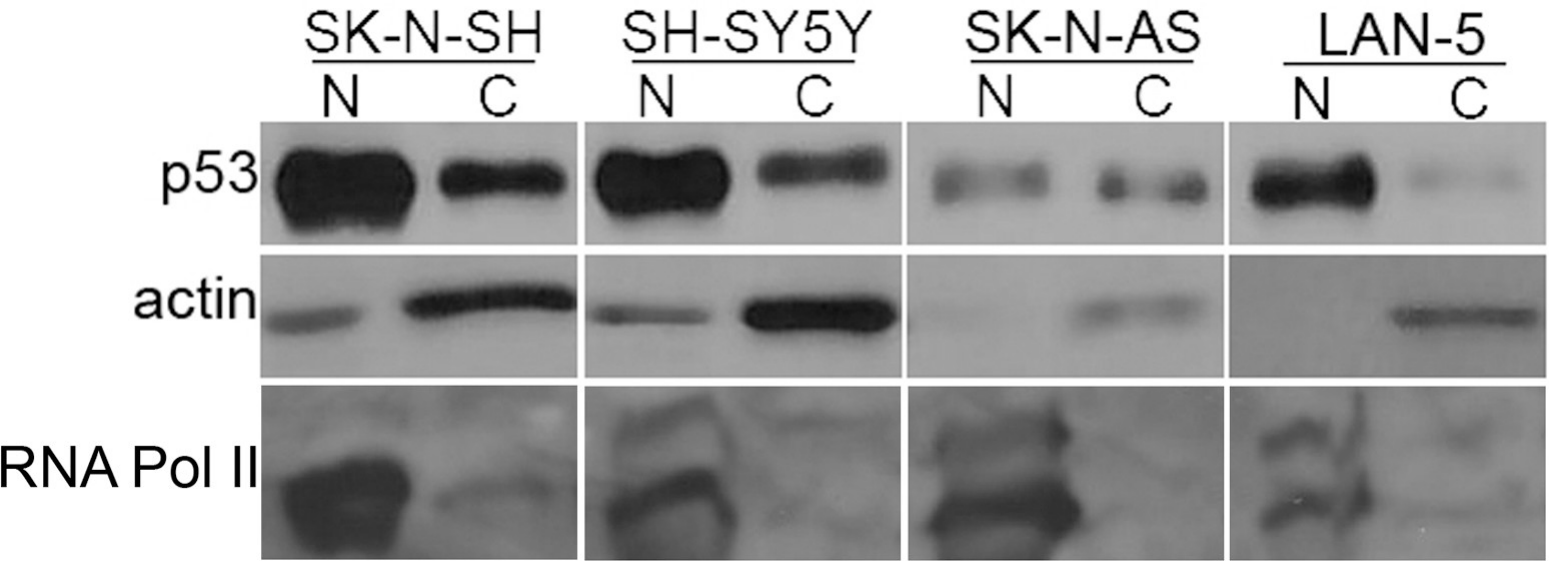
Nuclear and cytoplasmic distribution of p53 in SK-N-SH, SH-SY5Y, SK-N-AS, and Lan-5. Cells were treated with 10 ng/ml of SN38 for 24 h. Cells were harvested and nuclear and cytoplasmic extracts were prepared and analyzed by Western blot with p53-, actin-, or RNA Polymerase II-specific antibodies as indicated. All samples were run on the same gel, and lanes have been rearranged in final figure to ensure consistent ordering of cell lines in each figure.

Cytoplasmic sequestration has been proposed as one mechanism of inactivation of wildtype p53 in neuroblastomas [26, 27]. To determine whether that was the case in these cell lines, we prepared nuclear and cytoplasmic extracts and analyzed levels of p53 protein using Western blot. In all four cell lines, actin is largely found in the cytoplasmic extract and RNA Polymerase II is largely found in the nuclear extract as expected. In all cell lines except the SK-N-AS, p53 is largely found in the nuclear extract; in the SK-N-AS it is evenly split between the nuclear and cytoplasmic extracts (Fig 4). Thus, cytoplasmic sequestration does not explain the apparent defect in p53 function in the SH-SY5Y and Lan-5.

**Fig 5:**
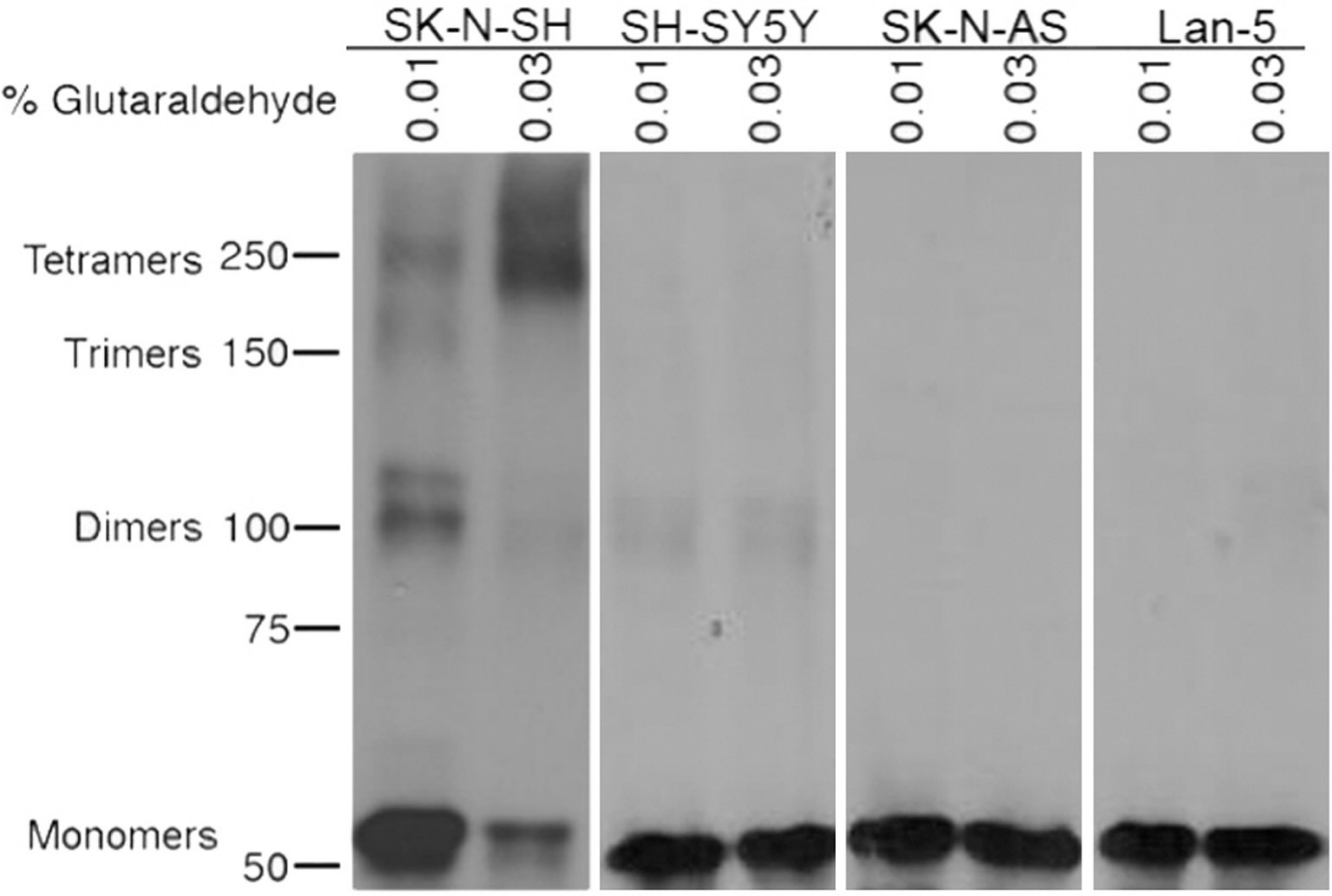
Analysis of p53 oligomerization in SK-N-SH, SH-SY5Y, SK-N-AS, and Lan-5. Cells were incubated with 10 ng/ml SN38 for 24 h. Cells were lysed and treated with 0.01 and 0.03% glutaraldehyde for 5 min. The lysates were then analyzed by Western blotting with p53-specific antibodies. All samples were run on the same gel, and lanes have been rearranged in final figure to ensure consistent ordering of cell lines in each figure.

In order to determine whether p53 oligomerization is defective in any of the cell lines, we performed glutaraldehyde crosslinking of SN38-treated lysates. SK-N-SH showed strong evidence of tetramerization at higher glutaraldehyde concentrations in addition to some dimer formation (Fig 5). This is similar to what has been observed in the p53-wildtype MCF10A cell line [36]. The SH-SY5Y cell line displayed no tetramers and very faint levels of dimers (Fig 5). These levels of dimers are similar to basal levels observed previously in non-SN38 treated cells [36]. The SK-N-AS and Lan-5 cell lines did not have any detectable levels of p53 oligomers (Fig 5).

## Discussion

Neuroblastomas are the most common extra-cranial tumors in infants and in children. It represents 8–10% of all tumors and 15% of all cancer-related deaths in children [1]. The prognosis is poor, with less than 50% survival rate [2–6]. Interestingly, p53 mutations are rare in neuroblastomas when compared to other cancers, with less than 2% of neuroblastomas containing p53 mutations [41]. Despite the wildtype p53 status of these tumors, the DNA damage-induced checkpoint response has been reported to be impaired in neuroblastomas through cytoplasmic sequestration of p53 [26, 27], although this remains controversial [31].

The use of DNA damaging agents and Chk1 inhibitors to overcome DNA damage-induced cell cycle arrest has been extensively studied as a strategy for cancer chemotherapy [14, 16, 17]. This method has largely been shown to be effective in cancers with defective p53, leaving p53-wildtype tumors such as neuroblastomas apparently off the table for this therapeutic combination. However, in our previous studies we have shown that some p53-wildtype tumors are sensitive to checkpoint abrogation mediated by the Chk1 inhibitor UCN-01[24]. In the current study, we investigated the response of p53-wildtype neuroblastoma tumors to this therapeutic combination.

In order to determine the response of neuroblastoma cells to DNA damage alone, SK-N-SH, SH-SY5Y, SK-N-AS, and Lan-5 cells were treated with varying concentrations of SN38 for 24 h. The cell lines arrested in a dose dependent manner, culminating in G1/S at the highest concentrations for SK-N-SH, SH-SY5Y, and Lan-5, similar to many p53-wildtype cell lines such as the immortalized breast epithelial cell line MCF10A [35]. However, the SK-N-AS arrested in G2 at the highest concentrations indicating an apparent defect in the G1/S checkpoint. It was surprising to find in the flow cytometry results that SK-N-SH appear to have a large increase of sub-G_1_ cells after treatment with intermediate SN38 concentrations. However, this result was not consistently seen in all trials, and may not be a meaningful observation (see Fig 3 for comparison).

In order to observe changes in levels of p53 and its activation, Western blot analysis was performed. The p53 levels rose with increasing SN38 in all four cell lines indicating p53 stabilization. The levels of p-p53-ser15 and p-p53-ser20 also increased, indicating that p53 was activated in response to DNA damage in all four cell lines. However, only the SK-N-SH had an increase in levels of p21^waf1^, consistent with normal p53 transactivation function. These cells were also the only ones observed to repress cyclin B. This was not the case with the SH-SY5Y, SK-N-AS, and Lan-5 cell lines, which had little to no p21^waf1^ induction and impaired repression of cyclin B. This is consistent with results we have previously observed in cells lacking functional p53 [35]. Taken together, the activation of p21 ^waf1^ and repression of cyclin B in SK-N-SH and not in the other three cell lines indicates that the former has normal p53 transcriptional activation and repression functions, whereas the latter do not.

When these cells were treated with the combination of SN38 followed by UCN-01, all but the SK-N-SH abrogated cell cycle arrest. Thus the SK-N-SH behave as expected for a p53-wildtype cell line, whereas the SH-SY5Y, SK-N-AS, and Lan-5 behave similarly to p53-defective cell lines. This suggests that the latter cell lines have another defect in p53 function not reflected in its mutational status. Although it has been suggested that neuroblastoma cell lines sequester p53 in the cytoplasm as an explanation of apparent p53 defects in p53-wildtype neuroblastomas [26, 27], we found no such cytoplasmic enrichment, with p53 enriched in the nucleus in all but the SK-N-AS, where the p53 was evenly distributed between the nuclear and cytoplasmic fractions. This is consistent with the observations of others that p53 is predominantly nuclear in neuroblastomas [31–33]. This observation prompted us to explore oligomerization as an alternate explanation for the lack of normal p53 function in some of the neuroblastoma cell lines.

We have previously analyzed the impact of p53 status on the cellular response to the combination of SN38 and UCN-01 using the p53-wildtype breast epithelial cells, MCF10A, as well as two isogenic derivatives: MCF10A/OD which overexpress the oligomerization domain as well as the MCF10A/Δp53 which overexpress an shRNA to p53 that targets p53 mRNA for degradation [35]. While the MCF10A were resistant to UCN-01-mediated abrogation of DNA damage-induced arrest, the MCF10A/Δp53 abrogated S and G2 arrest. Surprisingly, the MCF10A/OD cells abrogated S phase arrest but did not abrogate G2 arrest. This was explained by the fact that those cells failed to activate p21^waf1^, but still repressed cyclin B, indicating that the transcriptional activation and repression functions of p53 are distinct. Further studies revealed that the reason for the different response between the MCF10A/Δp53 and the MCF10A/OD were that the former failed to form any oligomers, while the latter failed to form tetramers, but still formed dimers [36]. We also observed that the presence of dimers was sufficient for binding of p53 to a putative head-to-tail response element in the cyclin B promoter. Thus, tetramers are essential for the transcriptional activation function of p53 but not for the repression function.

This led us to question whether the reason that some of the p53-wildtype neuroblastomas were sensitive to this combination while others were not could be explained by the absence or presence of p53 oligomers. Consistent to our expectations, only the SK-N-SH possessed p53 dimers and tetramers at levels approximating that of MCF10A [36]. The SH-SY5Y, SK-N-AS, and Lan-5 possessed little to no oligomers consistent with levels we have seen in the MCF10A/Δp53 or in untreated MCF10A [36].

Taken together, our results suggest that cytoplasmic localization does not serve as a reliable indicator for the likelihood that p53-wildtype neuroblastomas would be effectively treated with a therapeutic combination of a DNA damaging agent and a Chk1 inhibitor, but the oligomerization status may. Further studies are needed in order to determine the precise reason for the defect in oligomerization. Several proteins have been found to enhance p53 tetramerization, including BCCIP, members of the 14-3-3 family, and MYBBPIA, while others have been found to inhibit tetramerization, including RBEL1A, ARC [42]. Thus it is possible that the SH-SY5Y, SK-N-AS, and Lan-5 either overexpress one of the latter, underexpress one of the former, or both. Further studies will hopefully shed light on the p53 defects in neuroblastomas and open further avenues for therapeutic efficacy.

## Supporting information

S1 Fig Distribution of cell populations from two-dimensional flow cytometry analysis from SN38 dose responses. The percentage of cells in each quadrant of the two-dimensional flow cytometry: upper left (UL), lower left (LL), upper right (UR), and lower right (LR) for all six treatment conditions (SN38, 0, 0.3, 1, 3, 10, and 30 ng/ml), presented as mean ± SEM, n = 3. See Fig 1 for corresponding flow cytometry images.
(TIF)

S2 Fig Cell cycle distribution from UCN-01 treatments. The percentage of cells in each cell cycle stage (SubG1, G1, S, and G2/M) for each treatment condition presented as mean ± SEM, n = 3. See Fig 3 for corresponding flow cytometry images.
(TIF)

S1 File Raw images for Western blots. These images consist of original unmodified scans of Western blot films.
(PDF)
