# Supplementary figures and images for: p53 oligomerization status as an indicator of sensitivity of p53-wildtype neuroblastomas to the combination of DNA damaging agent and Chk1 inhibitor
Source: PLoS One. 2022 Feb 10;17(2):e0263463. doi: 10.1371/journal.pone.0263463 (PMC8830664; doi:10.1371/journal.pone.0263463)

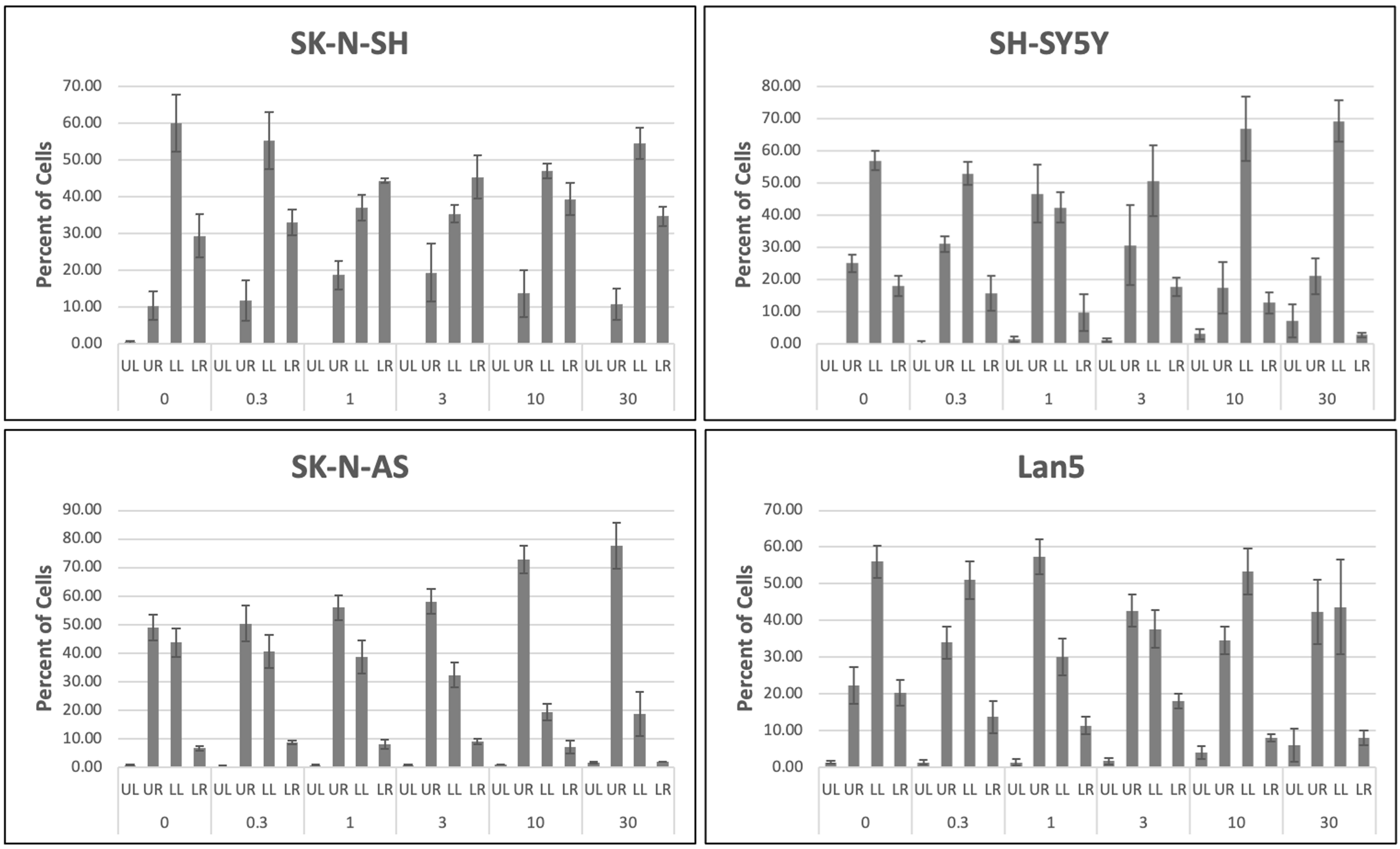

Supplement: S1 Fig — The percentage of cells in each quadrant of the two-dimensional flow cytometry: upper left (UL), lower left (LL), upper right (UR), and lower right (LR) for all six treatment conditions (SN38, 0, 0.3, 1, 3, 10, and 30 ng/ml), presented as mean ± SEM, n = 3. See Fig 1 for corresponding flow cytometry images. (TIF) [file pone.0263463.s001.tif]

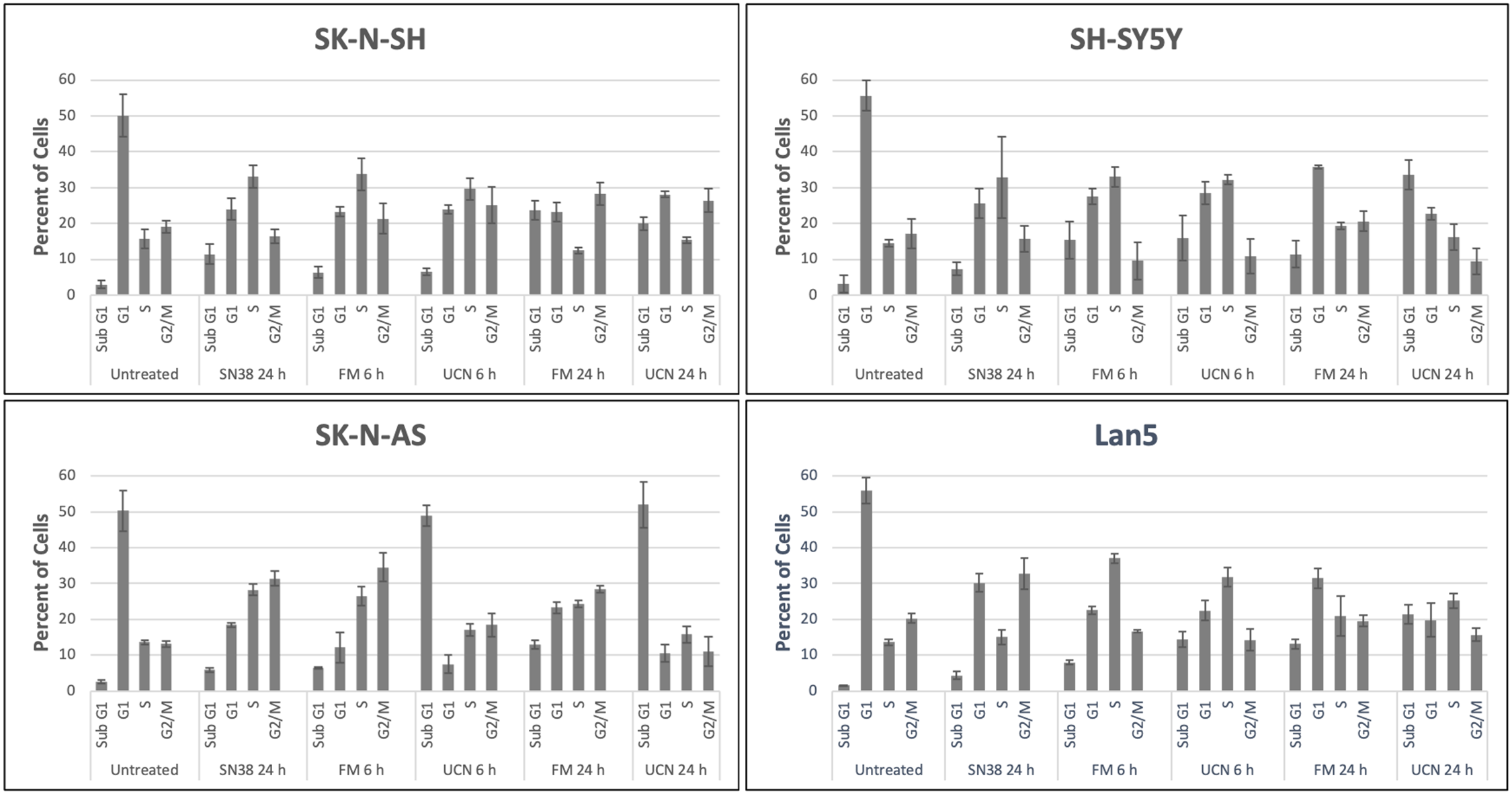

Supplement: S2 Fig — The percentage of cells in each cell cycle stage (SubG1, G1, S, and G2/M) for each treatment condition presented as mean ± SEM, n = 3. See Fig 3 for corresponding flow cytometry images. (TIF) [file pone.0263463.s002.tif]
