# Supplementary material for: p53 oligomerization status as an indicator of sensitivity of p53-wildtype neuroblastomas to the combination of DNA damaging agent and Chk1 inhibitor
Source: PLoS One. 2022 Feb 10;17(2):e0263463. doi: 10.1371/journal.pone.0263463 (PMC8830664; doi:10.1371/journal.pone.0263463)
Supplement: S1 File — These images consist of original unmodified scans of Western blot films. (PDF) [file pone.0263463.s003.pdf]

Nuclear and Cytoplasmic Extracts from Figure 4

For each antibody, all samples were run on the same gel; images were rearranged in the final figure in order to maintain consistent order of cell lines in all figures. Membranes were cut just above the 75 kDa marker to below the 36 kDa marker prior to antibody incubation. Blots were imaged on film which was hand-developed; after development film was aligned with the membrane in the cassette and the molecular weight ladder was marked by hand.

SK-N-AS SK-N-SH SH-SY5Y Lan-5
N C N C N C N C

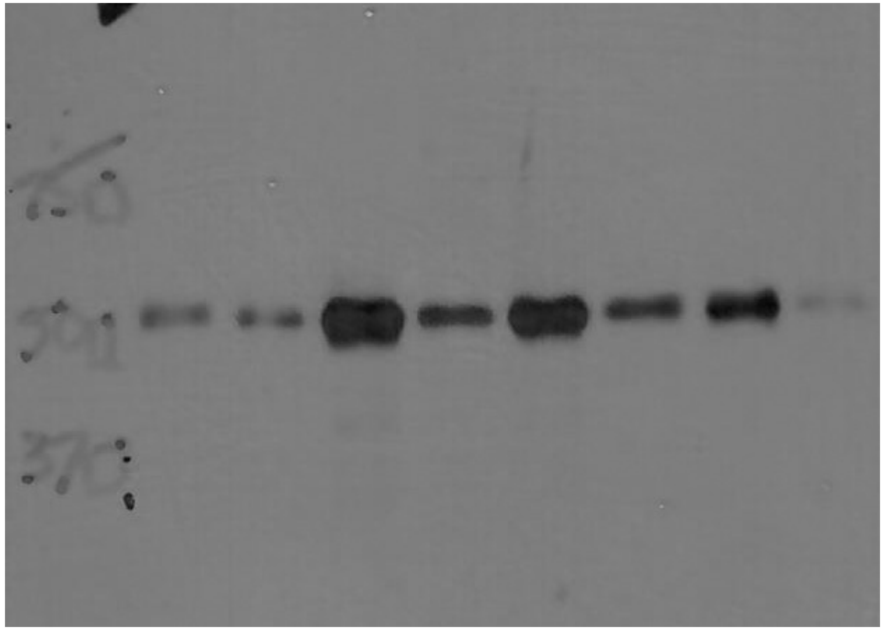

p53 Blot

1 min Exposure

Lan-5 SH-SY5Y SK-N-AS SK-N-SH
N C N C N C N C

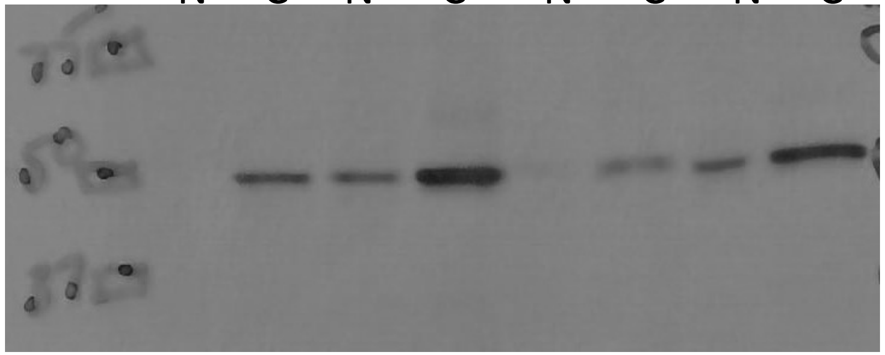

actin Blot

30 sec Exposure

Lan-5 SH-SY5Y SK-N-AS SK-N-SH
N C N C N C N C X X

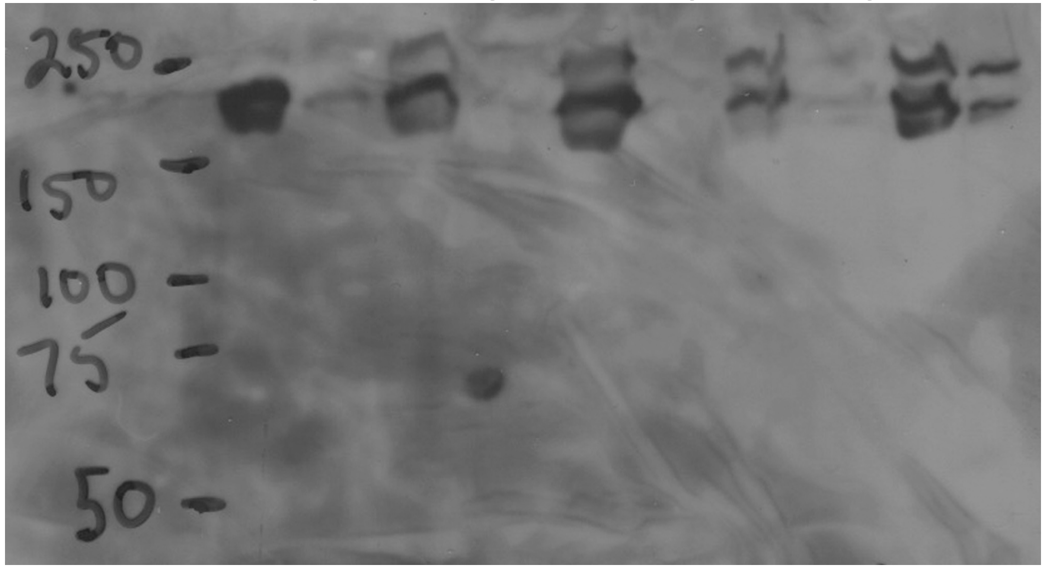

RNA Polymerase II Blot

1 min Exposure

Glutaraldehyde crosslinking from Figure 5

All samples were run on the same gel; images were rearranged in the final figure in order to maintain consistent order of cell lines in all figures.
Blots were imaged on film which was hand-developed; after development film was aligned with the membrane in the cassette and the molecular weight ladder was marked by hand.

SK-N-AS Lan-5 SH-SY5Y SK-N-SH

0.01 0.03 0.01 0.03 0.01 0.03 0.01 0.03 % Glutaraldehyde

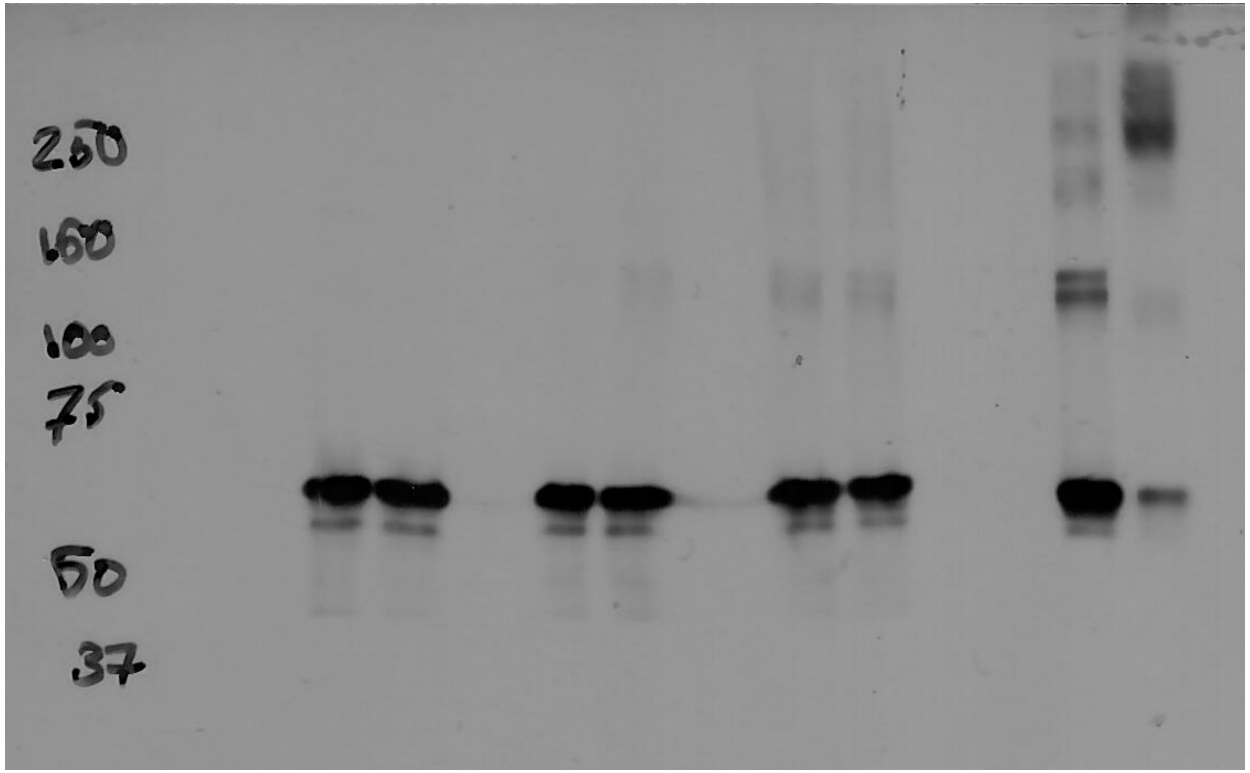

p53 Blot

1 Minute Exposure

SN38 Dose Response, Figure 2 (next 5 pages)

Given the number of cell lines it was not possible to consistently run all samples on the same gel. However, whenever possible samples were run on the same gel, and the same exposures were used for all blots using the same antibody.

In most cases membranes were cut just above the 50 or 75 kDa marker to below the 37 kDa marker prior to antibody incubation to conserve antibody (for p21 the membrane was cut below the 20 kDa and above the 37 kDa marker). Blots were imaged on film which was hand-developed; after development film was aligned with the membrane in the cassette and the molecular weight ladder was marked by hand.

The first two lanes, marked by X, are 0 and 30 ng/ml treated MCF10A cells.

SK-N-SH

0 30 | 0 0.3 1 3 10 30 ng/mL SN38

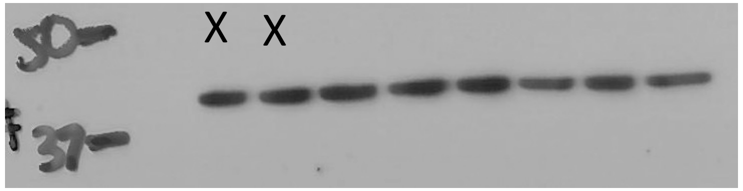

Actin, 30 sec exposure

SH-SY5Y

0 30 | 0 0.3 1 3 10 30 ng/mL SN38

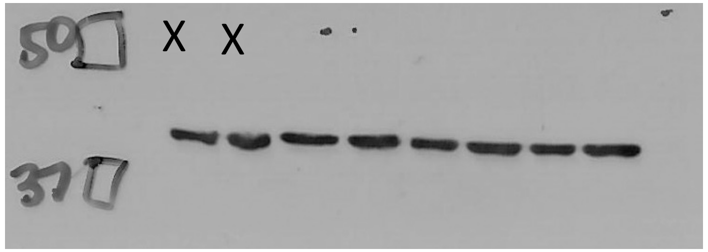

Actin, 30 sec exposure

SK-N-AS

0 30 | 0 0.3 1 3 10 30 ng/mL SN38

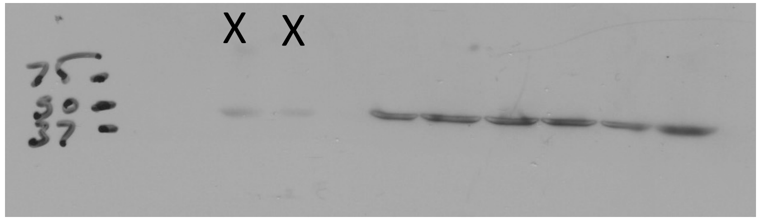

Actin, 30 sec exposure

Lan-5

0 30 | 0 0.3 1 3 10 30 ng/mL SN38

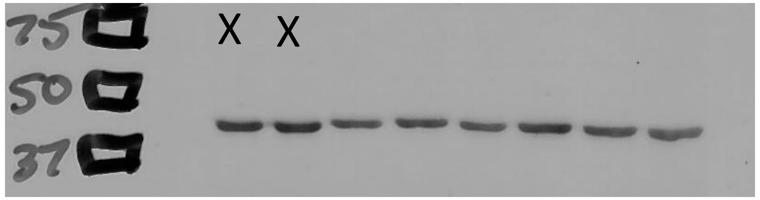

Actin, 30 sec exposure

SN38 Dose Response, Figure 2 (cont)

SK-N-SH						SH-SY5Y						
0	0.3	1	3	10	30	0	0.3	1	3	10	30	ng/mL SN38

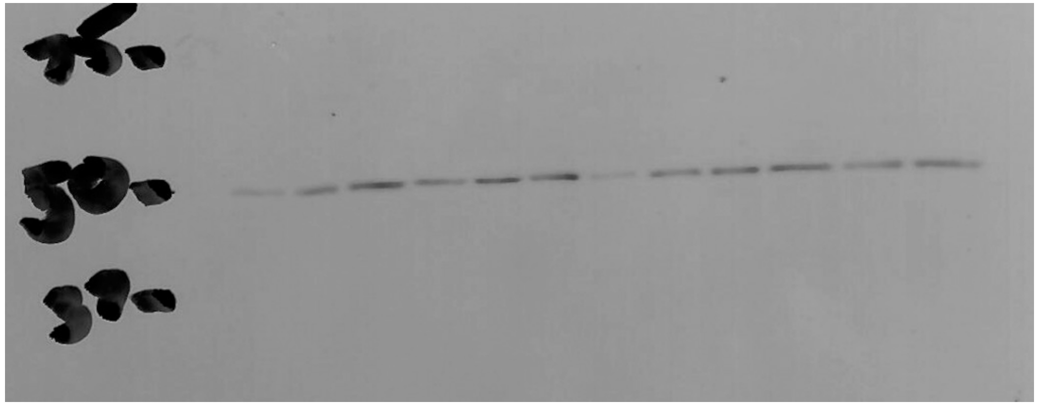

p53, 30 sec exposure

MCF10A		SK-N-AS						
0	30	0	0.3	1	3	10	30	ng/mL SN38
X	X							

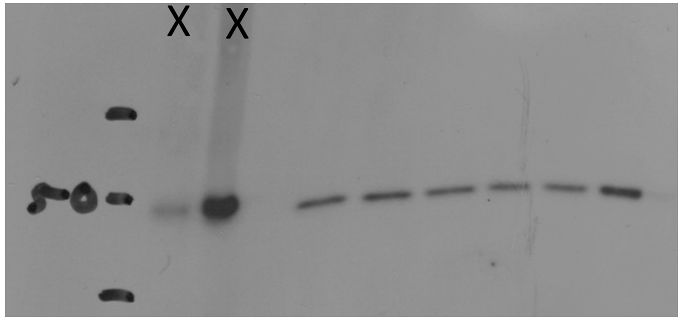

p53, 30 sec exposure

Lan-5						
0	0.3	1	3	10	30	ng/mL SN38

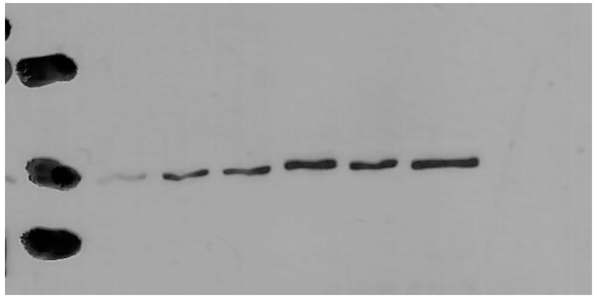

p53, 30 sec exposure

SN38 Dose Response, Figure 2 (cont)

MCF10A | SH-SY5Y | SK-N-AS

0 30 | 0 0.3 1 3 10 30 | 0 0.3 1 3 10 30 ng/mL SN38

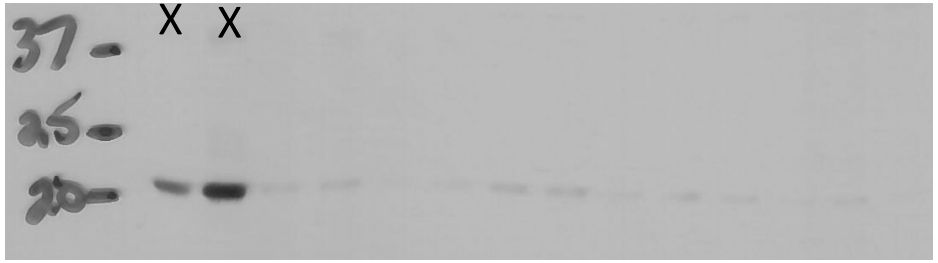

p21, 2 min exposure

MCF10A | Lan-5

0 30 | 0 0.3 1 3 10 30

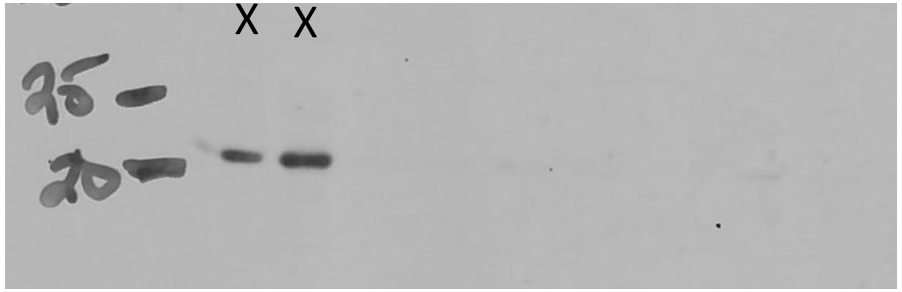

p21, 2 min exposure

SK-N-SH

0 0.3 1 3 10 30 ng/mL SN38

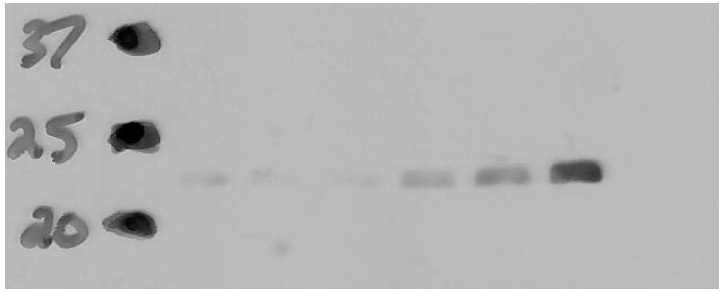

p21, 2 min exposure

SN38 Dose Response, Figure 2 (cont)

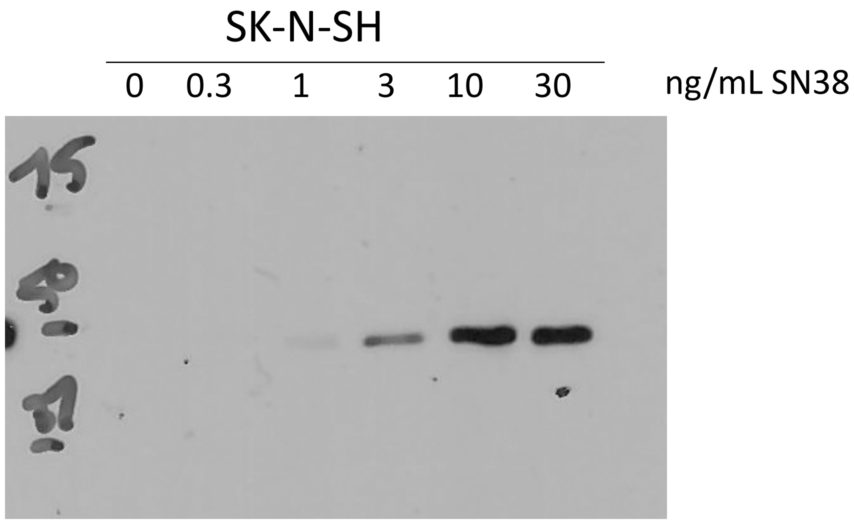

P-p53 (ser15), 2 min exposure

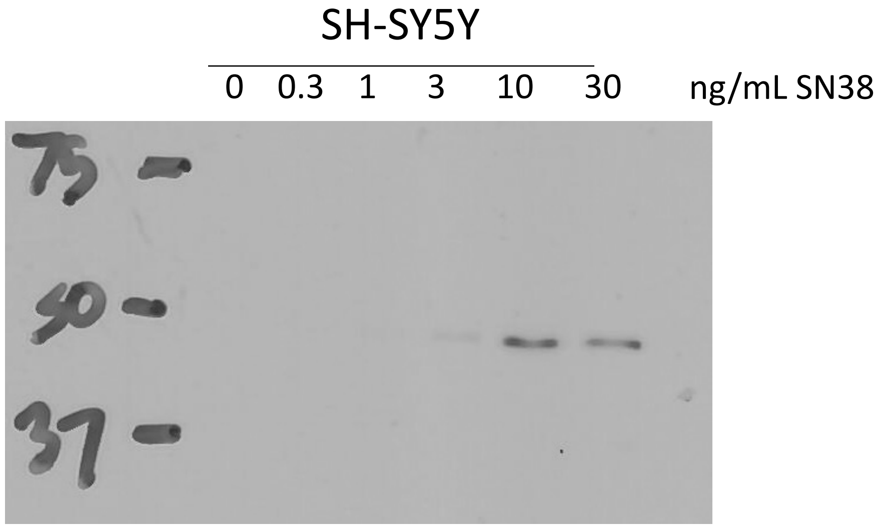

P-p53 (ser15), 2 min exposure

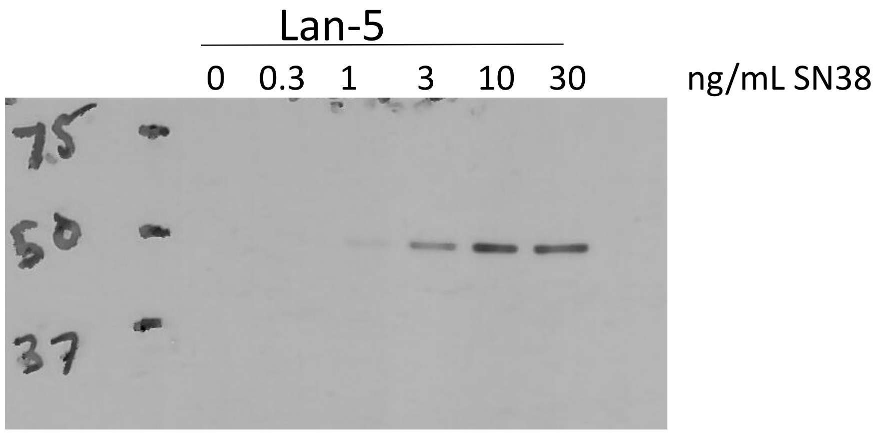

P-p53 (ser15), 2 min exposure

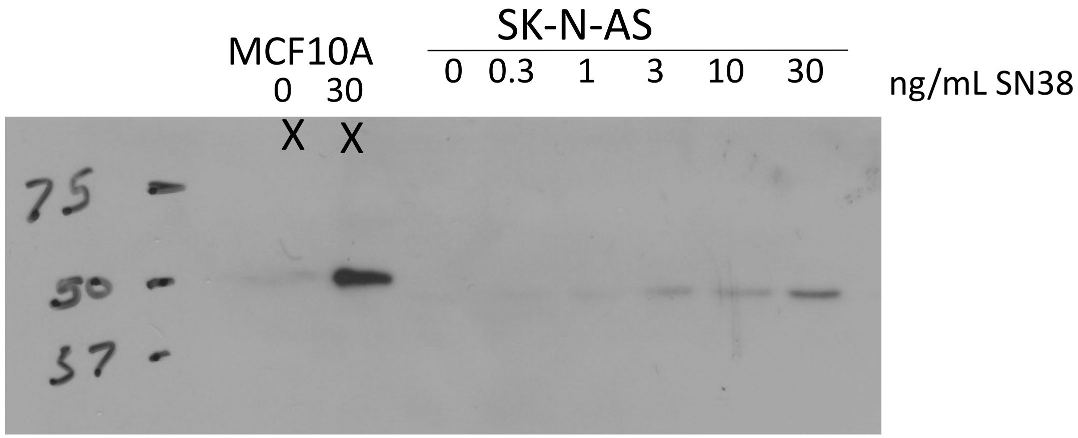

P-p53 (ser15), 2 min exposure

SN38 Dose Response, Figure 2 (cont)

SK-N-SH | SH-SY5Y

0 0.3 1 3 10 30 0 0.3 1 3 10 30 ng/mL SN38

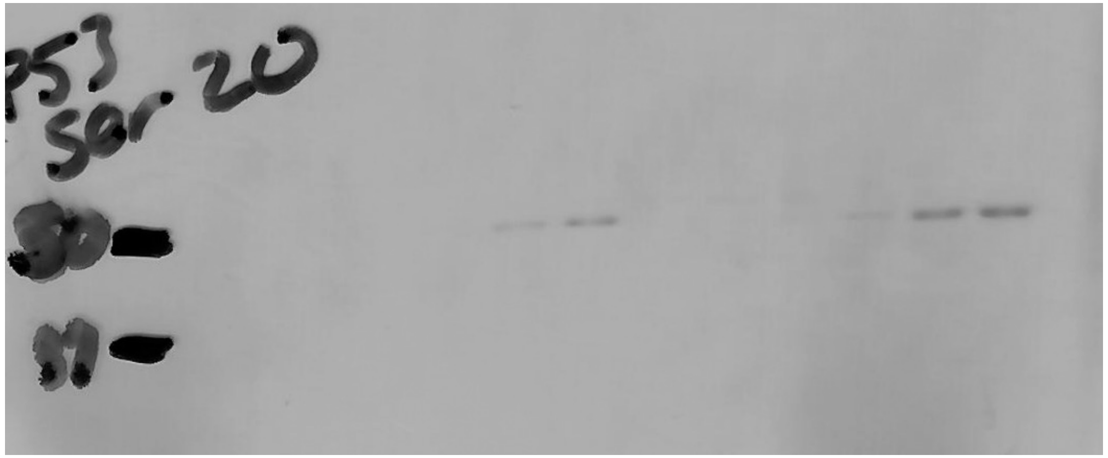

P-p53 (ser20), 2 min exposure

SK-N-AS | Lan-5

0 0.3 1 3 10 30 0 0.3 1 3 10 30 ng/mL SN38

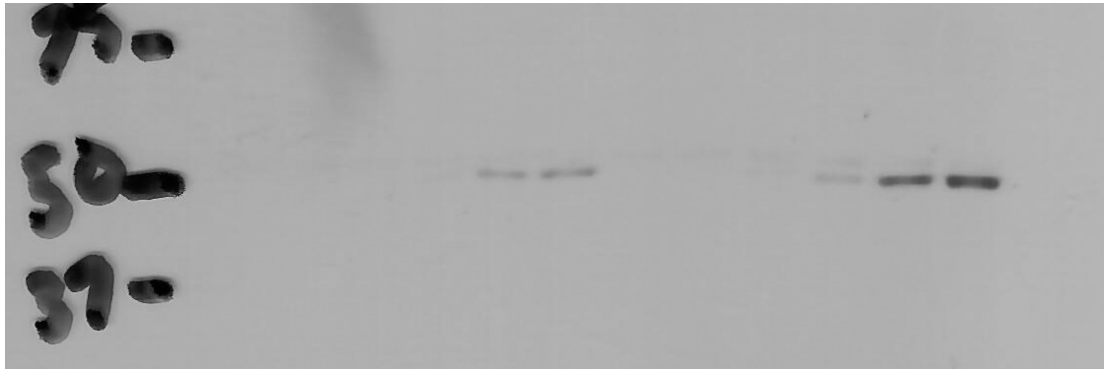

P-p53 (ser20), 2 min exposure
